# A Novel Mutator-Like Transposable Elements With Unusual Structure and Recent Transpositions in Barley (*Hordeum vulgare*)

**DOI:** 10.3389/fpls.2022.904619

**Published:** 2022-05-23

**Authors:** Dongying Gao, Ann M. Caspersen, Gongshe Hu, Harold E. Bockelman, Xianming Chen

**Affiliations:** ^1^Small Grains and Potato Germplasm Research Unit, USDA-ARS, Aberdeen, ID, United States; ^2^Wheat Health, Genetics, and Quality Research Unit, USDA-ARS, Pullman, WA, United States

**Keywords:** barley, mutator, genome evolution, horizontal transfer of transposon, ORFR

## Abstract

Mutator-like transposable elements (MULEs) represent a unique superfamily of DNA transposons as they can capture host genes and cause higher frequency of mutations in some eukaryotes. Despite their essential roles in plant evolution and functional genomics, MULEs are not fully understood yet in many important crops including barley (*Hordeum vulgare*). In this study, we analyzed the barley genome and identified a new mutator transposon *Hvu_Abermu*. This transposon is present at extremely high copy number in barley and shows unusual structure as it contains three open reading frames (ORFs) including one ORF (ORF1) encoding mutator transposase protein and one ORF (ORFR) showing opposite transcriptional orientation. We identified homologous sequences of *Hvu_Abermu* in both monocots and dicots and grouped them into a large mutator family named *Abermu*. *Abermu* transposons from different species share significant sequence identity, but they exhibit distinct sequence structures. Unlike the transposase proteins which are highly conserved between *Abermu* transposons from different organisms, the ORFR-encoded proteins are quite different from distant species. Phylogenetic analysis indicated that *Abermu* transposons shared closer evolutionary relationships with the maize *MuDR* transposon than other reported MULEs. We also found phylogenetic incongruence for the *Abermu* transposons identified in rice and its wild species implying the possibility of horizontal transfer of transposon. Further comparison indicated that over 200 barley genes contain *Abermu*-related sequences. We analyzed the barley pan genomes and detected polymorphic *Hvu_Abermu* transposons between the sequenced 23 wild and cultivated barley genomes. Our efforts identified a novel mutator transposon and revealed its recent transposition activity, which may help to develop genetic tools for barley and other crops.

## Introduction

Transposons or transposable elements (TEs) are mobile DNA sequences that have the capability to move from one location to another in genome(s). Transposons may induce harmful even lethal mutations that affect host fitness including some genetic diseases in humans (Payer and Burns, 2019). However, they may also cause chromosomal recombination, form new genes, and change gene expression. Therefore, transposons are considered an important driving force in gene and genome evolution (Kazazian, 2004). Transposons are classified into two major classes based on their transposition mechanisms and sequence structures. Class I elements or RNA retrotransposons mobilize *via* a copy-and-paste model and have the potential to dramatically increase copy number, whereas class II elements or DNA transposons transpose *via* multiple mechanisms including cut-and-paste, rolling circle replication and self-synthesizing transposition (Kapitonov and Jurka, 2006). Both class I and II elements can be further grouped into autonomous and non-autonomous transposons. The former are usually intact elements and encode necessary protein(s) for transposition, whereas the latter mostly contain internal deletions or accumulated mutations, do not generate functional transposases and their movement is catalyzed by the autonomous partners.

The mutator-like transposable elements (MULEs) are one major superfamily of DNA transposons, they were originally discovered in maize (*Zea mays*; Robertson, 1978) and have since been found in a very wide range of organisms including plants, animals, fungi and diatoms (Chalvet et al., 2003; Feschotte and Pritham, 2007). Typical MULEs contain relative long (can be over 300-bp) terminal inverted repeats (TIRs) and generate target site duplications (TSDs) of 7–12 bp (Yu et al., 2000). It has been revealed that some MULEs called Pack-MULEs have captured functional genes or fragments of expressed genes in both plants and animals (Yu et al., 2000; Jiang et al., 2004; Tan et al., 2021). In some cases, a Pack-MULE can contain fragments from multiple genes that may form new open reading frames (ORFs) and play unique roles in gene evolution (Jiang et al., 2004). Additionally, mutator elements may represent the most mutagenic plant transposons identified thus far as they insert into the maize genome at very high frequency (Lisch, 2002). Therefore, mutator transposons provide valuable resources for developing genetic tools to mutate genes and understand their molecular functions (Fernandes et al., 2004).

Although MULEs are widely present in eukaryotes, the vast majority of these elements were inactive and silent in host genomes due primarily to mutations, deletions and/or the epigenetic regulations on transposons (Singer et al., 2001; Lisch, 2009). Thus far, only a handful of active mutators have been identified including *Hop* in the fungus *Fusarium oxysporum* (Chalvet et al., 2003), *Os3378* in rice (*Oryza sativa*; Gao, 2012), *Muta1* in mosquito (*Aedes aegypti*; Liu and Wessler, 2017) and three elements in maize, *Mu9/MuDR* (Hershberger et al., 1991), *Jittery* (Xu et al., 2004) and *Mu13* (Tan et al., 2011). Except *Mu13*, all these active MULEs are autonomous transposons, but they show different sequence features. *Hop*, *Os3378*, *Muta1* and *Jittery* only have one gene encoding mutator transposase protein (Chalvet et al., 2003; Xu et al., 2004; Gao, 2012; Liu and Wessler, 2017). However, the *MuDR* transposon contains two genes including *mudrA* encoding the transposase MURA and *mudrB* encoding the help protein MURB (Lisch, 2002). Impressively, both *Os3378* and *Muta1* were able to transpose in yeast (*Saccharomyces cerevisiae*; Zhao et al., 2015; Liu and Wessler, 2017) suggesting their potential applications in distantly related species and the complex transposition and regulation mechanisms of MULEs. To better understand the evolution and transposition mechanisms of MULEs and to develop new resources for plant functional genetics, it is important to identify more new MULEs.

Barley (*Hordeum vulgare*, 2*n* = 2X = 14) is an important food and feed crop grown in a wide range of areas spanning from temperate to tropical climates. It has been cultivated for about 10,000 years and is one of the first domesticated crops (Badr et al., 2000). Barley belongs to the Triticeae tribe of the grass family which also contains bread wheat (*Triticum aestivum*, 2*n* = 6X = 42). Due to its diploid genome structure, close evolutionary relationships with the polyploidy wheat, and other unique features, barley has emerged as a model system for characterizing molecular functions of agronomically important genes in cereal crops (Carciofi et al., 2012). Thus far, over 20 genomes of cultivated barley and its wild progenitor, *Hordeum spontaneum*, have been sequenced and high repetitive fractions (over 80%) were detected in these genomes (Mascher et al., 2017; Jayakodi et al., 2020; Liu et al., 2020; Schreiber et al., 2020; Zeng et al., 2020; Xu et al., 2021). MULEs were identified in both cultivated and wild barley genomes (Mascher et al., 2017; Liu et al., 2020; Xu et al., 2021). However, the transposition and evolution of mutator transposons in barley is still poorly understood. In this study, we comparatively analyzed the barley genomes, discovered a new mutator transposon and investigated its transposition activity and evolution.

## Materials and Methods

### Plant Materials

A total of 11 cultivated barley varieties/landraces and five wild barley (*H. spontaneum*) accessions were collected in this study. The detailed information about the collected materials is shown in Supplementary Table 1. Five seeds for each genotype were planted, and the seedlings were grown in the greenhouse at the ARS Small Grains and Potato Germplasm Research Unit in Aberdeen, Idaho. The young leaves were collected from about 3-week seedlings, ground in extraction buffer with the 2010 Geno/Grinder (SPEX SamplePrep, Metuchen, NJ), and genomic DNA was extracted using the cetyltrimethyl ammonium bromide (CTAB) method (Doyle, 1990).

### Transposon Annotation

To identify MULEs in barley, the MURA transposase protein in maize (accession no. AAA21566) was first used to search against the barley full-length cDNAs (FLcDNAs) database (Matsumoto et al., 2011), the significant hits (E-value <1 × e^−10^) then served as the queries to conduct BLASTN searches against the version 2 pseudomolecules_assembly^1^ of the six-row malting barley cultivar “Morex” (Mascher et al., 2017), hereafter referred to as the barley reference genome. The significant hits and their 20-Kb flanking sequences (10-kb for each side) were extracted and used to define complete mutator transposons based on the terminal inverted repeats and target site duplications. The complete mutator element identified in barley was then used to search against GenBank and find homologous transposons in other genomes. We combined two approaches for predicting transposon structures and transposase proteins: (1) the internal region of each complete transposon was annotated by Fgenesh (Solovyev et al., 2006) and GENSCAN (Burge and Karlin, 1997); and (2) the internal region was also used to conduct BLASTX search and to predict the gene structures based on the homologous proteins deposited in GenBank. In addition, we also searched against the rice genome annotation database^2^ and other public genome annotation websites to gain more insights into the transposon structures.

### Detection of Polymorphic Transposons

The representative 8,176-bp complete transposon was applied to screen the barley reference genome (Mascher et al., 2017) with RepeatMasker program^3^ using the default parameters. The “nolow” option was selected to avoid masking the low-complexity DNA. All hits were manually inspected to identify complete transposons and typical derivative elements and determine their exact boundaries based on the TIRs, flanking TSDs and sequence comparisons with the representative element. The 600-bp flanking sequence (300-bp for each side) for each transposon with well-defined boundaries was used to search against the genomes of 20 cultivated barley varieties and two wild barley accessions (Liu et al., 2020; Jayakodi et al., 2020; Schreiber et al., 2020; Zeng et al., 2020; Xu et al., 2021) and to detect its presence/absence in other 22 barley genomes based on the sequence alignment.

### Identification of Mutator-Related Genes and Sequence Alignment

The barley reference genome and the gene annotations (GFF3 file; Mascher et al., 2017) were used to extract the full gene sequences including both exons and introns with the bedtools package.^4^ The representative complete element was used to search against both CDS database and the extracted gene sequences in barley to detect if any genes contain transposon-related sequences. All significant hits (E-value < 1 × e^−10^) were then manually inspected, and the redundant hits were removed.

To compare the transposon-related gene in barley with the orthologous regions in wild barley and bread wheat, the gene and the 20-Kb flanking region were extracted and used to conduct BLASTN searches against the corresponding regions in the wild species and wheat with the default options, except that we applied the output eight format. The sequence alignments were conducted with the Artemis Comparison Tool (ACT) program.^5^

### Estimation of Non-synonymous and Synonymous Substitution Rates

The protein sequences of mutator transposons and alcohol dehydrogenases 1 (ADH1) were aligned with ClustalW using MEGA11 software (Tamura et al., 2021), and the alignment of multiple protein sequences and the cDNA sequences were used to calculate the number of non-synonymous substitutions per non-synonymous site (Ka) and the number of synonymous substitutions per synonymous site (Ks) using the PAL2NAL program (Suyama et al., 2006). The ADH1 protein (accession no. ADH03830.1) in cultivated rice (*Oryza sativa*) was used as a query to search against GenBank and identify homologous sequences in other *Oryza* species.

### PCR Validation of Polymorphic Transposons

Both transposons and their flanking sequences (300-bp for each side) were obtained from the Morex genome and used to design primers with the Primer3 program (Untergasser et al., 2012). The designed primers (Supplementary Table 2) were synthesized by the Eurofins Genomics LLC (Louisville, KY, United States) and applied for PCR analysis. PCR amplifications were conducted in a Bio-Rad S1000 Thermal Cycler in 20 μl reactions consisting of ~50 ng of genomic DNA, 0.2 mM primer, deionized water, and 10 μl EconoTaq PLUS GREEN 2X Master Mix (Middleton, WI, United States) containing 0.1 units/μl of EconoTaq DNA Polymerase, Reaction Buffer (pH 9.0), 400 μM dATP, 400 μM dGTP, 400 μM dCTP, 400 μM dATP, and 3 mM MgCl_2_. The PCR temperature cycling conditions were 1 cycle of 98°C for 2 min; 35 cycles of 95°C for 30 s, 55°C for 30 s, 72°C for 30 s; and 1 cycle of 72°C for 5 min. Amplification products were run on 0.8% agarose gels and stained with ethidium bromide.

### Construction of Phylogenetic Trees

Both conserved domains and entire protein sequences of mutator transposases were aligned using ClustalW program with default options and the ambiguous regions were trimmed. The alignments of multiple sequences were then used to generate evolutionary trees with software MEGA11 (Tamura et al., 2021) using the neighbor-joining method. The phylogenetic analysis was performed based on 1,000 bootstrap replicates.

## Results

### Identification of a New Mutator Transposon

To identify putative transcriptional mutator transposons in barley, the maize *MuDR* transposase protein, MURA, was used as a query to search against the barley full-length (FL) cDNA database (Matsumoto et al., 2011). Eight significant hits (E-value <1 × e^−26^) were found and a FLcDNA sequence (accession no. AK251670) stood out as it showed the lowest E-value (6 × e^−82^). AK251670 was then used to search against the barley genome sequences (Mascher et al., 2017) and to find complete mutator transposons according to the TIRs and TSDs. An 8,176-bp transposon was identified, sequence searches indicated that its DNA exhibited no significant identity to any described MULEs. We named the new element *Hvu_Abermu*. This element contains the TIRs of 138–145 bp and is flanked by 9-bp TSD. The relative long TIRs and 9-bp TSD are similar to the typical features of other MULEs including *Hop* (Chalvet et al., 2003), *Os3378* (Gao, 2012), *MuDR* (Hershberger et al., 1991), *Jittery* (Xu et al., 2004) and *Mu13* (Tan et al., 2011). However, *Hvu_Abermu* was much larger than these MULEs that ranged in size from 1,495 bp (*Mu13*) to 4,942 bp (*Os3378*).

**Figure 1:**
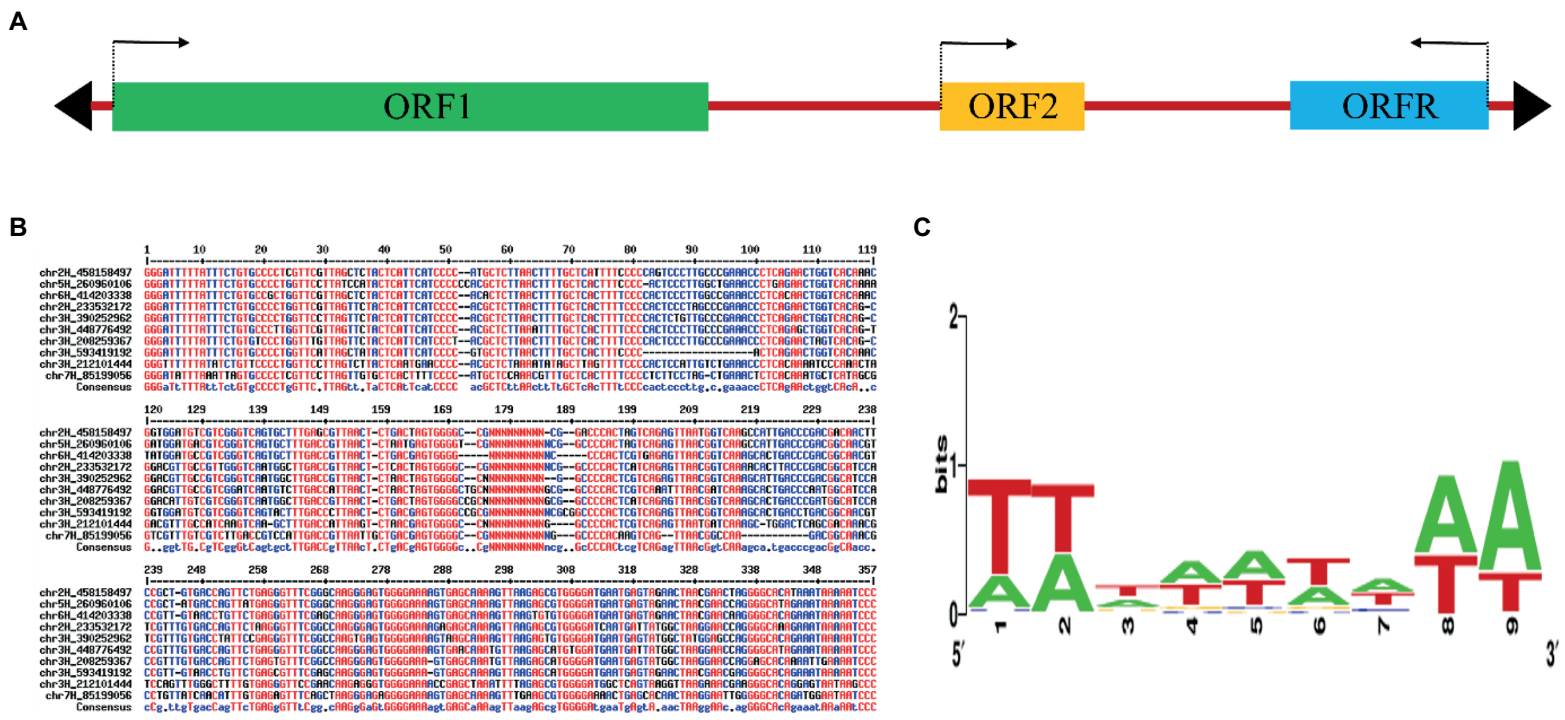
A mutator transposon in barley. **(A)** The sequence structure of *Hvu_Abermu*. Colorful rectangles and black triangles represent the open reading frames (ORFs) and terminal inverted repeats (TIRs) of the transposon and the arrows indicate the transcription orientations. **(B)** Sequence alignment of 5′ and 3′ TIRs of 10 *Hvu_Abermu* transposons. The internal regions of transposons are indicated by 20 Ns. **(C)** WebLogo of 9-bp TSDs of 267 *Hvu_Abermu* elements in barley.

The typical *MuDR* transposon contains two genes with transcription occurring in opposite directions, whereas many other autonomous MULEs only have one gene encoding mutator transposase protein. Impressively, *Hvu_Abermu* contains three open reading frames (ORFs), and the first two ORFs, ORF1 and ORF2, showed the same direction of transcription. However, the third ORF, ORFR, is transcribed in the opposite orientation compared to ORF1 and ORF2 (Figure 1A). The 826-aa protein encoded by ORF1 exhibited significant sequence similarity to MURA (E-value = 2 × e^−62^) and other mutator transposase proteins. Significant hits (E-value <1 × e^−10^) of ORF2 were identified in barley, wheat, *Brachypodium distachyon*, *Dichanthelium oligosanthes*, *Panicum virgatum* and wild relatives of wheat. However, the function of ORF2-coding protein is not clear. Despite both *mudrB* of maize *MuDR* transposon and the ORFR of *Hvu_Abermu* showed opposite direction of transcription to the ORF encoding mutator transposase, they shared no sequence identity at nucleotide or protein levels. Since the protein encoded by ORF1 of *Hvu_Abermu* shows similar size to MURA, and also contains complete transposase domains including DDE and zinc finger (ZnF) that are essential for catalyzing mutator transposition, *Hvu_Abermu* is likely an autonomous MULE.

### High Copy Number and Insertion Preference

Like other DNA transposons that mobilize with a cut-and-paste mechanism, many mutator families are present at low copy number in host genomes. For example, the *Jittery* transposon is present in maize inbred lines at zero to two copies (Xu et al., 2004). The barley reference genome was screened using RepeatMasker and identified 11,430 repeats including both complete elements and deletion derivatives. Usually, one repeat including the fragmental element caused by insertion or deletion can be counted as one copy of *Hvu_Abermu*. However, in some cases (less than 10% occurrence), one transposon was split into two or more repeats. Thus, we estimated there are about 10,000 *Hvu_Abermu* copies in the barley genome including complete transposons, truncated elements, or fragmental sequences derived from *Hvu_Abermu*. It is worth noting that this number may be underestimated as the barley genome is highly repetitive and about 5% of the genome sequences were not assembled into the reference genome (Mascher et al., 2017).

The *Hvu_Abermu* sequences and the flanking regions were subsequently manually inspected. The vast majority of these elements were fragmented and lacked TIRs and TSDs. However, the exact boundaries for 278 *Hvu_Abermu* transposons were identified based on their TIRs and TSDs including 267 elements flanked by 9-bp TSDs, 10 elements surrounded by 8-bp TSDs and one transposon flanked by 10-bp TSDs. No TSD was found for six *Hvu_Abermu* transposons, but their boundaries can be clearly determined by the conserved 5′GGG…CCC3′ terminal motifs of *Hvu_Abermu* elements (Figure 1B) and sequence comparisons with the 8,176-bp reference *Hvu_Abermu* suggesting that either the TSDs have undergone dramatic mutations and cannot be recognized, or their insertions did not generate TSD as some MULEs did (Liu and Wessler, 2017). Our sequence inspection indicates that *Hvu_Abermu* frequently generated 9-bp TSDs when they integrated into the barley genome. The flanking 9-bp TSDs of the 267 *Hvu_Abermu* transposons were extracted and aligned, the multiple sequence alignments suggest that the *Hvu_Abermu* transposons preferentially insert into TA-rich regions (Figure 1C).

**Figure 2:**
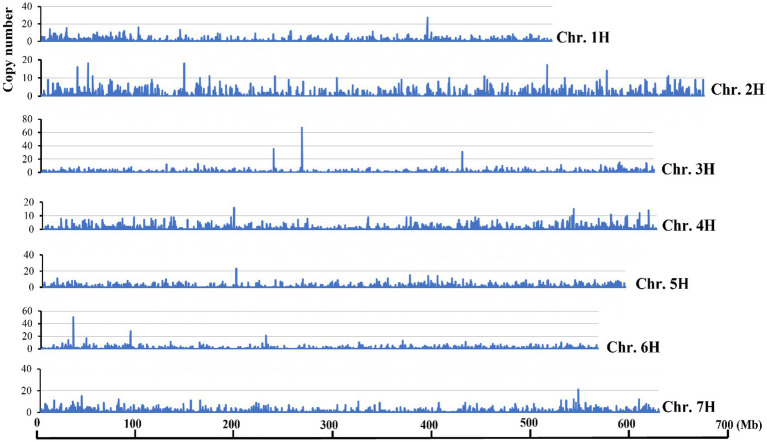
Distributions of *Hvu_Abermu* transposons on seven barley chromosomes.

Transposons showed uneven distributions across the barley genome and the chromosome termini have accumulated/retained more DNA transposon sequences than other regions (Mascher et al., 2017). To test if *Hvu_Abermu* transposons showed similar trends, the copy numbers for each 200-Kb window was counted and along with the estimated density of *Hvu_Abermu* elements. The results indicate that *Hvu_Abermu* transposons were dispersed across the barley genome and no higher density of transposons was detected in the terminal regions of seven barley chromosomes (Figure 2). However, variable densities among the seven chromosomes were observed. The average transposon density was 2.65 *Hvu_Abermus* per Mb sequence for the seven chromosomes. Chromosome 1 had the highest density (2.85 transposons/Mb), whereas chromosome 4 had the lowest density (2.18 transposons/Mb).

### *Abermu* Family Transposons are Present in Both Monocots and Dicots

Transposons are genomic sequences that evolve very rapidly (Fedoroff, 2012). Therefore, homologous transposons are difficult to identify in distantly relative species (Gao et al., 2009). To detect if the homologous sequences of *Hvu_Abermu* are present in other genomes, GenBank was searched with the 8,176-bp *Hvu_Abermu* sequence. Significant hits were identified in 71 flowering plants (Angiosperms) including 66 monocots and five dicots, but no homolog was found in other main divisions of land plants. The hit sequences and their 20-Kb flanking regions (10Kb for each side) were extracted and manually inspected. We identified complete transposons in 38 flowering plants including 36 species of the family Poaceae, one non-grass monocot (*Carex littledalei*) and one dicot (*Sedum album*; Table 1). As these elements shared significant sequence identity to *Hvu_Abermu* and between each other, *Hvu_Abermu* and the homologous transposons were grouped into the same family called *Abermu*.

**Table 1:** List of *Abermu* transposons in plants.

Transposon	Host species/family	Location	Target site duplication
*Ata_Abermu*	*Aegilops tauschii*/Poaceae	MCGU01055324:836238-844306	TAGAAGAAA
*Bdi_Abermu*	*Brachypodium distachyon*/Poaceae	ADDN03000002:2433447-2448826	TACTTGATA
*Cam_Abermu*	*Cenchrus americanus*/Poaceae	LKME02052029: 131820544-131816611	AATTTAATA
*Caq_Abermu*	*Coix aquatica*/Poaceae	RZMS01000001:139462938-139467897	TAATAAGAA
*Cla_Abermu*	*Coix lacryma-jobi*/Poaceae	VDCT01000002:38534105-38539063	TAATAAGAA
*Ecr_Abermu*	*Echinochloa crus-galli*/Poaceae	JADDKO010000091: 1996190-2000480	ATTTTCAAA
*Eco_Abermu*	*Eleusine coracana*/Poaceae	LXGH01512356:31259-26712	TATTATAAA
*Ein_Abermu*	*Eleusine indica*/Poaceae	QEPD01000594:105014-108678	TAAATTTAA
*Ecu_Abermu*	*Eragrostis curvula*/Poaceae	RWGY01000039:33855193-33859169	CTAAAAAAT
*Eni_Abermu*	*Eragrostis nindensis*/Poaceae	JAAXCT010000717:7467-10289	AATGTAAAA
*Lpe_Abermu*	*Leersia perrieri*/Poaceae	ALNV02005249:51572-55878	AAATTGAAA
*Hvu_Abermu*	*Hordeum vulgare*/Poaceae	CAJHDE010025927:939984-948159	TTTGAAATA
*Hsp_Abermu* ^*^	*Hordeum spontaneum*/Poaceae	CAJRBJ010000345:64885-84128	TTCTAGAAA
*Oth_Abermu*	*Oropetium thomaeum*/Poaceae	LFJQ01000008:43819-38956	TTATCTTAA
*Obr_Abermu*	*Oryza brachyantha*/Poaceae	AGAT02000065:4032146-4036948	TTTTTGAAA
*Ogl_Abermu*	*Oryza glaberrima*/Poaceae	ADWL02000019: 3168286-3163128	AAAGTTGTA
*Ogr_Abermu* ^**^	*Oryza granulata*/Poaceae	RYFJ01000168:385548-393530	ATTCTATTT
*Olo_Abermu*	*Oryza longistaminata*/Poaceae	WNHE01001000: 16821562-16816427	TACTAGAAA
*Ome_Abermu*	*Oryza meridionalis*/Poaceae	LONC01001747:31486-36262	TAAAAATAA
*Oni_Abermu*	*Oryza nivara*/Poaceae	AWHD02000030: 2258628-2253824	TTTTGCAAT
*Opu_Abermu*	*Oryza punctata*/Poaceae	AVCL02000059: 7701364-7696327	TAACCATT
*Oru_Abermu*	*Oryza rufipogon*/Poaceae	LONB01002577:79564-74479	TATTTTATA
*Osa_Abermu*	*Oryza sativa*/Poaceae	CP056061:5936454-5941084	CATATATATATA
*Pha_Abermu*	*Panicum hallii*/Poaceae	QAVV01000028:931513-924701	TACTTTATT
*Pvi_Abermu*	*Panicum virgatum*/Poaceae	JABWAI010000356:32715-22565	TAATTAATA
*Pte_Abermu*	*Puccinellia tenuiflora*/Poaceae	QRDG01000350:895002-906340	ATTTTTAAA
*Ssp_Abermu*	*Saccharum spontaneum*/Poaceae	UINE01012842:75382-70169	TTGAAAGAA
*Sce_Abermu*	*Secale cereale*/Poaceae	JADQCU010000006:503716784-503725460	TTTGAAAAA
*Sit_Abermu*	*Setaria italica*/Poaceae	LWRS01000002:28051658-28046894	TCTACTTGCAC
*Svi_Abermu*	*Setaria viridis*/Poaceae	SNSE01000010:1381381-1377615	TCTACTTGCGT
*Tel_Abermu*	*Thinopyrum elongatum*/Poaceae	JAAAXO010000002:137000763-137009254	AATCATTAA
*Tae_Abermu*	*Triticum aestivum*/Poaceae	JAGHKL010000009:339035754-339043964	TAATTTTTA
*Tdi_Abermu*	*Triticum dicoccoides*/Poaceae	CACRSD010017326:108212-116532	AAAGATATA
*Tur_Abermu*	*Triticum urartu*/Poaceae	AOTI010647462:10206-1960	TTTTCTTTA
*Zja_Abermu*	*Zoysia japonica*/Poaceae	BCLF01000099:114106-118890	TTCTTTATT
*Zma_Abermu*	*Zoysia matrella*/Poaceae	BCLG01000797:100507-105405	ATTGACTAA
*Zmay_Abermu* ^**^	*Zea mays*/Poaceae	RAQR01000005:80660794-80645890	ATCACCAAA
*Cli_Abermu*	*Carex littledalei*/Cyperaceae	SWLB01000018:4805699-4813155	TTTTTTTTA
*Sal_Abermu*	*Sedum album*/Crassulaceae	QZGG01000138:248974-242462	TAATTTTTA

* Nested by a CACTA transposon;

** Nested by a LTR retrotransposon.

**Figure 3:**
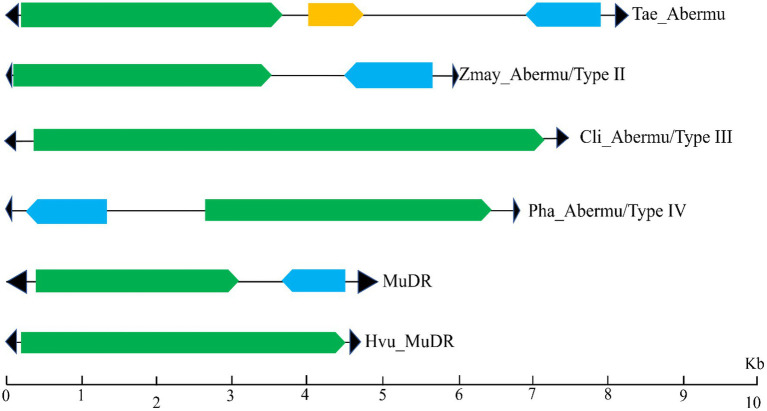
The sequence structures of different types of *Abermu* transposons and *MuDR* elements. The black triangles represent the TIRs of transposons. The green rectangles represent the ORF (ORF1) encoding mutator transposase protein, the blue rectangles indicate that ORF (ORFR) showing opposite orientation of transcription and the orange rectangle mean the second ORF that has the same transcription orientation with ORF1.

To test if *Abermu* elements in other plants exhibit similar structures with *Hvu_Abermu*, we annotated the representative elements in the 38 plants (Table 1) using Fgenesh (Solovyev et al., 2006) and GENSCAN (Burge and Karlin, 1997). These elements showed distinct structures that can be grouped into four types (Figure 3). The Type I *Abermu* elements showed the same structure as *Hvu_Abermu*, and their internal regions contain three ORFs. These transposons were only identified in the tribes Triticeae and Poeae including common wheat (*Triticum aestivum*, *Tae_Abermu*), *Aegilops tauschii* (*Ata_Abermu*), wild emmer wheat (*Triticum dicoccoides*, *Tdi_Abermu*), *Aegilops tauschii* (*Ata_Abermu*), *Hordeum spontaneum* (*Hsp_Aberm*) and *Puccinellia tenuiflora* (*Pte_Abermu*). The type II transposons which have two ORFs, the first ORF (ORF1) encodes mutator transposase protein, and the second ORF (ORFR) showed the opposite transcription direction to ORF1 and was located between ORF1 and 3′ TIR of the transposon. The type II *Abermu*s were found in 13 plants including maize (*Zea mays*, *Zmay_Abermu*), two *Coix* species, four *Oryza* species and three plants of the tribe Triticeae, rye (*Secale cereale*, *Sce_Abermu*), *Triticum urartu* (*Tur_Abermu*) and *Thinopyrum elongatum* (*Tel_Abermu*). The type III transposons only have one ORF encoding mutator transposase, this type of *Abermu* was discovered in 17 plants including five *Oryza* species, two *Eragrostis* species, two *Setaria* species and two non-grass plants, *Carex littledalei* and *Sedum album*. In addition, we also found that *Abermus* in three plants, *Panicum hallii* (*Pha_Abermu*), *Panicum virgatum* (*Pvu_Abermu*) and *Brachypodium distachyon* (*Bdi_Abermu*), showed structures that were different from the three types above. For example, *Pha_Abermu* contains an opposite ORF (ORFR) located between 5′ TIR and the transposase-encoded ORF (ORF1).

### Divergent ORFR Sequences

**Figure 4:**
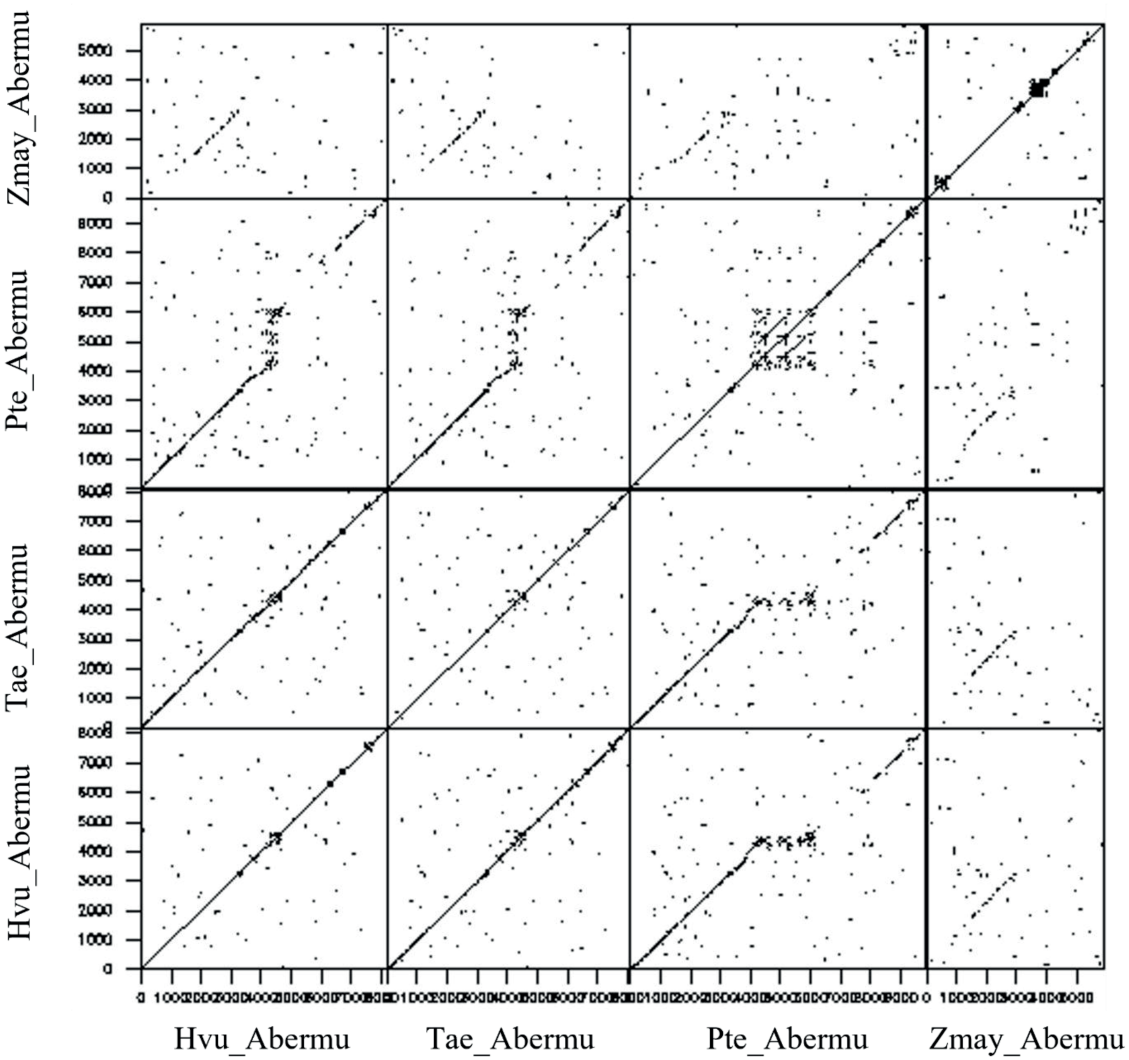
Dotplot analysis between *Hvu_Abermu* and three other *Abermu* transposons in bread wheat, *Puccinellia tenuiflora* and maize.

We further compared the *Abermu* elements that contain both ORFR and the ORF encoding mutator transposase (ORF1) to gain clues about their sequence divergence and relationships. Our sequence comparisons indicated that the ORF1 regions of these *Abermu* transposons showed significant sequence identity between each other. However, the ORFR regions were very dynamic, and no sequence similarity was detected for some elements. For example, the ORFR sequence of *Hvu_Abermu* only shared similarity with the *Abermu*s identified in the Triticeae and Poeae including *Tae_Abermu*, *Sce_Abermu* and *Pte_Abermu* but lacked detectable similarity with the ORFRs of *Zmay_Abermu* (Figure 4) and the elements identified in *Oryza* and other genomes. Thus, these comparisons suggested that the ORFR sequences tended to be less conserved than the ORF1 regions. In maize, *Zmay_Abermu* exhibited similar structure with *MuDR* transposon (Figure 3). However, the DNA sequence of *Zmay_Abermu* shared no sequence identity to *MuDR*, implying that they have diverged from an ancestor mutator for a long evolutionary period.

Previous studies revealed that some genomic regions have diverged more rapidly than others as they may have been subjected to different levels of selective pressures (Davidson et al., 2009; Seehausen et al., 2014; Sendell-Price et al., 2020). To gain insight into the sequence divergence of *Abermu*s and test if different ORFs of the transposons have undergone similar levels of selective constraint, the number of non-synonymous substitutions per non-synonymous site (Ka) and synonymous substitutions per synonymous site (Ks) of three ORFs were calculated by comparing *Hvu_Abermu* with the three *Abermu* elements in related species. The Ka/Ks ratios for all three ORFs were less than 1 (Table 2) suggesting that they have undergone purifying selection. The Ka and Ks values of three pairs of ORF1 were similar to that of ORF2. However, the Ka and Ks values of ORFR were much higher than both ORF1 and ORF2, and the average Ka and Ks values were three to five times larger than that of ORF1 and ORF2 (Table 2), implying that ORFR regions have likely been subjected to relatively looser selection pressure than ORF1 and ORF2.

**Table 2:** Ka and Ks values of three ORFs between *Hvu_Abermu* and its three homologs.

*Hvu_Abermu*		*Tae_Abermu*	*Ata_Abermu*	*Tdi_Abermu*
ORF1	Ka	0.0347	0.1148	0.0964
	Ks	0.0556	0.1533	0.1234
	Ka/Ks	0.6239	0.7494	0.7810
ORF2	Ka	0.0759	0.1125	0.0934
	Ks	0.1011	0.1874	0.1209
	Ka/Ks	0.7507	0.6000	0.7727
ORFR	Ka	0.6075	0.2281	0.1269
	Ks	1.3499	0.4074	0.3288
	Ka/Ks	0.4500	0.5598	0.3858

### Phylogenetic Analysis of *Abermu* Transposons

**Figure 5:**
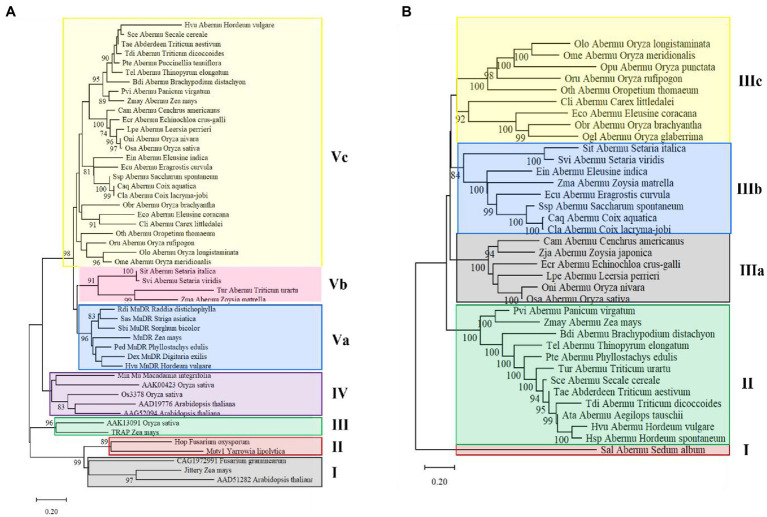
Phylogenetic analysis of *Abermu* transposons and other mutator elements. **(A)** The phylogenetic tree made with the conserved domain of mutator transposase. **(B)** The phylogenetic tree built using whole mutator transposase. The branches with over 70% bootstrap values are marked.

To understand the evolutionary origin of *Abermu* transposons, phylogenetic analysis was performed using the conserved domain of mutator transposase proteins of 30 complete *Abermu* elements from different plants (Table 1) and 19 MULEs published or identified in this study (Supplementary Table 3). Eight *Abermu* transposons listed in Table 1 were excluded from this analysis as their transposases lacked the conserved domain sequences or contained large deletions. The 49 MULEs were grouped into five clades, *Jittery*, *Os3378* and *Hop* fell into different clades which were distantly related to *Abermu* transposons (Figure 5A). All *Abermu* transposons formed two subclades within the clade V sister to the subclade of *MuDR*-related elements in plants. Thus, *Abermu* transposons shared closer relationship with *MuDR* than other reported MULEs. The *Abermu* transposons identified in closely related species were mostly grouped together, but we found that the *Abermu*s in six *Oryza* species were distantly separated.

As the conserved domains of the mutator transposase proteins were short [about 100 amino acids (aa)] and may only provide clues about the sequence divergence in the core regions of MULEs but not for the whole encoding regions. Thus, we further generated a phylogenetic tree with the whole transposase proteins of all complete *Abermu* transposons (Table 1) but *Eni_Abermu*, *Ogr_Abermu* and *Pha_Abermu* as these three encode shorter proteins (<420-aa). Other MULEs including *Hop* from a fungus was also excluded as their protein sequences were too divergent and can cause problematic alignment regions leading to more biased topologies (Talavera and Castresana, 2007). *Sal_Abermu* in the dicot *Sedum album* was separated from all 35 *Abermu* transposons found in monocots which fell into two clades. All *Abermu* transposons in the tribes Triticeae and Poeae were grouped into the clade II, and the *Abermu*s in eight *Oryza* species were grouped into the two subclades of the clade III (Figure 5B). Consistent with the phylogeny of the conserved domains, *Cli*_*Abermu*s, identified in *Carex littledalei* of the family Cyperaceae, was closely related to *Eco_Abermu* in *Eleusine coracana* that belongs to grass family. Both phylogenetic trees built with the conserved domains and whole transposase proteins indicated the phylogenetic separations and incongruence of *Abermu*s from *Oryza* species as we found that the *Abermu* elements in rice and its wild species, *O. nivara*, showed close phylogenetic relationships to the *Abermu* from *Leersia perrieri* which is the outgroup species of the *Oryza* genus (Stein et al., 2018).

### *Abermu*-Related Genes in Barley

**Figure 6:**
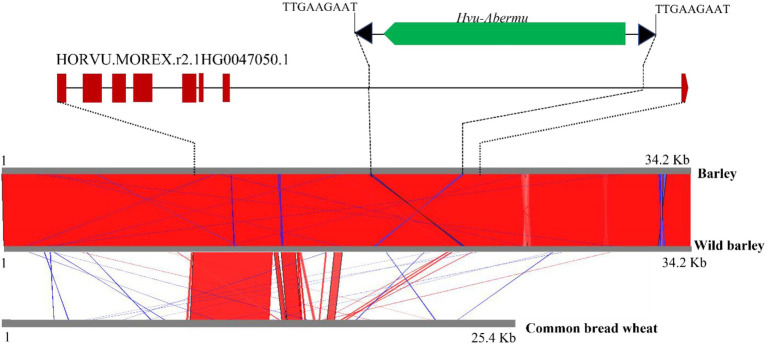
Sequence alignments of the orthologous genes and 20-Kb flanking regions in cultivated and wild barley and bread wheat. Top shows the structure of the barley *HORVU.MOREX.r2.1HG0047050.1* gene and the inserted *Hvu_Abermu* element. The brown rectangles/pentagon represent the exons of the barley gene, and the black solid lines mean introns of the gene. Bottom is the alignments of the orthologous genes and flanking regions. Red means shared sequences showing same orientations and blue means shared regions with reverse.

Transposons play important roles in gene evolution and morphological variation (Kazazian, 2004; Ong-Abdullah et al., 2015). Since *Abermu* elements are very abundant in the barley genome, we wonder if any *Abermu* elements were involved in gene formations. The annotated genes in the barley genome (Mascher et al., 2017) was searched using the *Hvu_Abermu* element as the query. The coding DNA sequences (CDSs) of 211 annotated genes showed significant sequence identity (E-value <1 × e^−10^) to *Hvu-Abermu*. Among them, seven genes encode mutator transposase protein and another 204 genes encode proteins with various molecular functions including metabolic pathways, disease resistance, and others (Supplementary Tables 4). Furthermore, *Hvu-Abermu* was found in the intronic regions of seven annotated genes including *HORVU.MOREX.r2.1HG0047050.1* that harbors a *Hvu-Abermu* with partially internal deletion in the seventh intron (Figure 6).

To gain clues about when this *Hvu-Abermu* element inserted into the gene, DNA sequence of *HORVU.MOREX.r2.1HG0047050.1* and the 20-Kb flanking regions (10-Kb each side) was extracted and searched against the genomes of the wild barley (*H. spontaneum*) B1K-04-12 (Jayakodi et al., 2020) and the bread wheat (Zhu et al., 2021). The homologous sequences were detected for both the gene including the *Hvu-Abermu* and the 20-Kb flanking region in wild barley (Figure 6) suggesting that *Hvu-Abermu* inserted into the barley genome before its domestication occurring about 10,000 years (Badr et al., 2000). Three homologous genes of *HORVU.MOREX.r2.1HG0047050.1* were identified in wheat genome, *TraesCS1A03G0605600.1*, *TraesCS1B03G0707700.1* and *TraesCS1D03G0582200.1*. However, no *Abermu* sequence was identified within all three genes and the flanking regions, even we searched against their 200-Kb surrounding regions implying that the *Hvu-Abermu* element likely inserted into ancient wild barley after the divergence of barley and wheat, or the element was also present in the original ancestor of wheat but has excised before the radiation of three diploid wild wheat species (AA, BB, and DD).

### Polymorphic Transposons in the Barley Pan Genomes

Potential active transposons can be identified based on the high sequence identity between the family members (Jiang et al., 2003). The complete *Hvu-Abermu* elements in barley was compared and found that they can share more than 95% sequence identity suggesting that *Hvu-Abermu* may be an active mutator. To detect its transpositional activity, the 600-bp flanking sequences (300-bp for each side) of the 284 *Hvu_Abermu* transposons with well-defined boundaries in the reference barley genome from the malting barley cultivar “Morex” (Mascher et al., 2017) were used to search against the genomes of 22 cultivated and wild barley accessions, including 18 cultivated barley genotypes and one accession of wild barley (B1K-04-12) generated by the barley pan-genome project (Jayakodi et al., 2020; Schreiber et al., 2020), one Canadian malting barley cultivar “AAC Synergy” (Xu et al., 2021), one Tibetan hulless barley “Lasa Goumang” (Zeng et al., 2020) and another wild barley accession AWCS276 (WB1; Liu et al., 2020). No hits or multiple highly identical hits were detected for the flanking sequences of 160 *Hvu_Abermu* transposons suggesting that these *Hvu_Abermu* elements were either inserted into highly repetitive regions or the flanking regions were not sequenced and/or assembled in the 22 genomes. However, the orthologous regions in the 22 genomes for 124 *Hvu_Abermu* elements were defined based on the sequence alignments. We found that 121 elements are shared by Morex and other barley genomes indicating that these *Hvu_Abermu* transposons have been retained in the population before the domestication of cultivated barley. Interestingly, three polymorphic *Hvu_Abermu* elements were identified that are present in the Morex genome but absent in some wild and barley genomes (Supplementary Table S5). For example, the *Hvu_Abermu* element located on chr7H (92741435–92758274) in Morex is absent in the wild barley B1K-04-12 and six cultivated barley accessions, HOR_7552, HOR_13942, Igri, OUN333, ZDM01467 and ZDM02064, but it is present in the wild barley WB1 and other 15 cultivated barley materials.

To detect more polymorphic *Hvu_Abermu* transposons, the 8,176-bp *Hvu_Abermu* was also used to screen the genomes of “Golden Promise” (Schreiber et al., 2020), “Lasa Goumang” (Zeng et al., 2020) and WB1 (Liu et al., 2020). We identified 278 complete *Hvu_Abermu* transposons and deletion derivatives with well-defined TIRs in the Golden Promise genome. We have extracted their 600-bp flanking sequences and used to compare with the Morex genome (Mascher et al., 2017). The orthologous loci for 119 *Hvu_Abermu* elements were identified in the Morex genome. Among them, 118 are shared between “Golden Promise” and “Morex.” However, one transposon (chr2H: 555341970–555347145) in “Golden Promise” is absent in “Morex.” In addition, the flanking sequence for 246 *Hvu_Abermus* transposons in “Lasa Goumang” was extracted and compared with the Morex genome. We found 108 *Hvu_Abermu* elements shared by “Lasa Goumang” and “Morex” and two *Hvu_Abermu* transposons located in the sequences DOW01000362.1 (860851–869592) and SDOW01000553.1 (879368–888197) present in “Lasa Goumang” but absent in “Morex.” In the WB1 genome, we extracted the flanking sequences of 284 *Hvu_Abermu* elements and searched against the Morex genome and found 117 *Hvu_Abermu* elements shared between WB1 and “Morex.” One *Hvu_Abermu* transposon (WB_00002568:81405–90263) is present in WB1 but absent in “Morex.” Taken all together, four *Hvu_Abermus* transposons were found that are present in one of the three genomes but absent in the Morex genome. We further detected the distributions of these four polymorphic elements in the barley pan genome and found these elements are absent in most of the 23 genomes (Supplementary Table S5).

**Figure 7:**
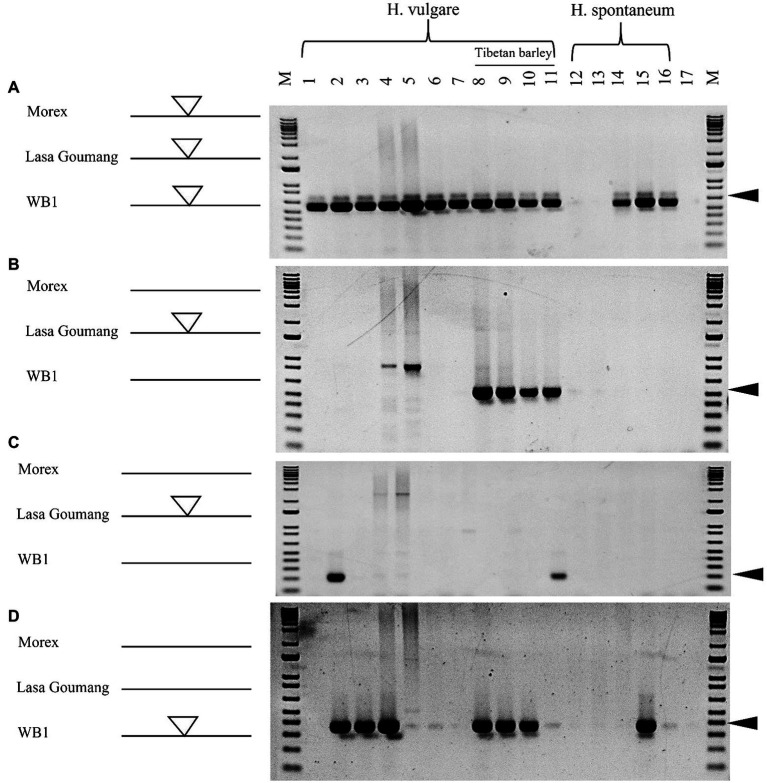
PCR analysis of 16 barley genotypes using the primers of UAAB5 **(A)**, UAAB11 **(B)**, UAAB13 **(C)**, and UAAB7 **(D)**. M means the 1-Kb plus DNA ladder. One to eleven represent 11 cultivated barley genotypes, 1: Morex; 2: Akashinriki; 3: Igri; 4: HOR_3081(Slaski); 5: Golden Promise; 6: Hockett; 7: AAC Synergy; 8: Qingki; 9: Tibetan (PI 506343); 10: Tibetan (PI 195542); 11: Tibetan (CIho 3,087), and the 12–16 indicated five wild barley accessions, 12: PI 282572; 13: PI 282574; 14: PI 282575; 15: PI 282588 and 16: PI 282593. Seventeen is a negative control and no DNA template was added for PCR amplification. The white and bigger triangles represent the inserted transposons in the left side, and the black and smaller triangles in the right side mean the expected sizes of amplifications.

To validate the computational analyses performed, 16 barley genotypes were collected for PCR analysis including “Morex,” “Golden Promise” and other five cultivated barley varieties that genomes have been sequenced by the barley pan-genome and other projects (Jayakodi et al., 2020; Schreiber et al., 2020; Xu et al., 2021). We were not able to obtain the seeds or DNAs for “Lasa Goumang” and two wild barley B1K-04-12 and AWCS276 (WB1) but we collected four Tibetan hulless varieties and five wild barley accessions from the National Small Grains Collection (NSGC) at the USDA (Supplementary Table 1). Four pairs of PCR primers were designed based on the *Hvu_Abermu* transposons and their flanking sequences. For each pair, one primer targets the flanking sequence, and the other anchors the *Hvu_Abermu* transposon, thus an expected amplicon will be generated if the transposon is present in the orthologous locus. Using the UAAB5 primers designed with a *Hvu_Abermu* transposon (chr3H:168627665_168630547) in “Morex,” an expected band was amplified in all 11 cultivated barley varieties and three wild accessions (Figure 7A), which confirmed our sequence analysis that showed this element is shared by “Morex” and the other 22 wild and cultivated barley genomes including “Golden Promise,” “AAC Synergy,” “Lasa Goumang” and the two wild accessions (Supplementary Table S5). Using the UAAB11 primers targeting the polymorphic *Hvu_Abermu* (DOW01000362.1: 860851–869592) in Lasa Goumang, an amplicon with expected size (530-bp) was only found in four accessions of Tibetan barley (Figure 7B), it was consistent with our genomic comparisons showing that this transposon is present in “Lasa Goumang” but absent in the two wild barley accessions, “Morex” and “Golden Promise.” We also amplified the DNAs of 16 barley samples using the UAAB13 primers targeting a *Hvu_Abermus* transposon (SDOW01000553.1:879368–888197) in “Lasa Goumang,” the specific band was only amplified in a Tibetan barley (CIho 3,087) and Akashinriki (Figure 7C) that confirmed its presence in “Akashinriki” and absence in “Morex,” “Golden Promise,” “AAC Synergy,” and other barley varieties. Our sequence comparisons identified a *Hvu_Abermu* (WB_00002568:81405–90263) in the wild barley WB1 and revealed that it is present in “Lasa Goumang,” “Akashinriki,” “Igri,” and “HOR_3081,” but absent in “Morex,” “Golden Promise,” “AAC Synergy,” “Hockett” and the wild barley B1K-04-12 (Supplementary Table S5). Using the UAAB7 primers targeting this polymorphic element in WB1 and its flaking region, an expected PCR band was amplified in “Akashinriki,” “Igri,” “HOR_3081,” three Tibetan barley accessions and one wild barley (PI 282588) but no band was amplified in “Morex,” “Golden Promise” and other two (Figure 7D). Our PCR experiment revealed polymorphic *Hvu_Abermu* transposons in 16 wild and cultivated barley accessions and the results exactly matched the presence/absence of the *Hvu_Abermu* transposons in the seven non-Tibetan barley varieties identified by our computational analysis. For the four Tibetan barley varieties and five wild barley accessions, the absence of transposons may need to be further validated with sequencing data as point mutations and other factors can cause the failure of PCR amplification.

## Discussion

### A New and Unusual MULE

In this study, a new mutator family *Abermu* was identified. Unlike other MULEs, *Abermu* transposons exhibit several unusual features in barley and its relatives. First, they are larger (over 8 Kb) and contain three ORFs including two extra ORFs with unknown functions, this type of transposon was not reported before. Second, they are present at extremely high copy numbers. We estimated that there are about 10,000 *Abermu* repeats in the barley genome and over 30,000 copies in the common wheat. MULEs transpose through a cut-and-paste mechanism and usually do not increase their copy numbers. It is not clear how *Abermu* transposons reached such high copies in the cereals. In *Drosophila*, the *P* elements can quickly increase their copy number *via* the gap repair mechanism or inserted into the chromosomes during S phase of meiosis (Spradling et al., 2011). Third, the insertion preference. Previous studies indicated that mutator transposons preferentially insert into genes in maize (Cresse et al., 1995; Fernandes et al., 2004). Our analysis suggests that *Abermu* are frequently inserted into TA-rich regions (Figure 1C). It seems that TA-rich regions play critical roles in plant gene expression as they may harbor the TATA box of promoters or other core motifs for gene regulation (Forde et al., 1990). Fourth, they demonstrated a possible higher retention rate. Transposon insertions may cause deleterious mutations and affect the host fitness, especially for the transposons with large sizes (Petrov et al., 2003). Therefore, transposons showed high turnover rates and can be quickly removed from the host genome. Genome-wide comparisons indicated that only 17 to 25% of full-length LTR in the wild barley B1K-04-12 are shared with the domesticated barley genotypes (Jayakodi et al., 2020). We analyzed the barley pan genomes and found that 98% (121/124*100) of complete *Abermu* elements are shared between two wild barley accessions and 21 cultivated barley genotypes (Supplementary Table S5). The retention rate of *Abermu* is three times or higher than the LTR retroelements.

### Complex Evolution of *Abermu* Transposons

Our phylogenetic analysis suggests that *Abermu* share more close evolutionary relationship with *MuDR* than other reported MULEs (Figure 5A). However, these two mutator families have likely diverged a long time ago as the DNA sequence of *Zmay_Abermu* exhibited no sequence identity to *MuDR* in maize. In addition, a mutator transposon in barley called *Hvu_MuDR* (Figure 3) was identified which showed 64% sequence identity (E-value = 2 × e^−53^) to *MuDR* in maize but no significant sequence identity to *Hvu_Abermu* transposon in barley. Therefore, both *Abermu* and *MuDR* may have existed in the common ancestor of maize and barley which split about 50 million years ago (Gaut, 2002). Given that *Abermu* transposons are found in both dicots and monocots (Table 1), the emergence of *Abermu* family likely occurred even earlier than the divergence of maize and barley. Like many other genomic sequences, transposons are most transferred from parent to offspring, vertical transfers of transposons (VTT). However, they can also be transferred horizontally between reproductively isolated species even between plants and animals (Gao et al., 2018). The shared *Abermu* elements between barley and its wild species (Figures 6, 7; Supplementary Table S5) indicated the vertical transfers of *Abermu* elements. We also detected phylogenetic separations of *Abermu* elements in different *Oryza* species and *Osa_Abermu* in rice was grouped together with *Lpe_Abermu* but not with the *Abermu* from *O. rufipogon*, the wild progenitor of cultivated rice (Figures 5A,B). As *L. perrieri* has diverged from the *Oryza* genus about 15–20 million years ago (MYAs; Stein et al., 2018) and transposons were difficult to be retained for a long period. The phylogenetic separation and incongruence imply the possibility of horizontal transfer of *Abermu* between *L. perrieri* and rice. The synonymous divergence rate (Ks) provides another clue for HTTs (Diao et al., 2006). We calculated the Ks values of *Abermu* and the class I alcohol dehydrogenase (*ADH1*) gene between *L. perrieri* and each of the seven *Oryza* species (Table 3). The Ks value of *ADH1* was similar for each pair of the comparisons. However, the Ks rates of *Lpe_Abermu/Osa_Abermu* and *Lpe_Abermu/Oni_Abermu* were much lower than other comparisons suggesting that *Lpe _Abermu* showed lower sequence divergence with *Osa_Abermu* and *Oni_Abermu* than with the *Abermu*s in other *Oryza* species. Thus, both phylogenetic analysis and sequence divergence suggest possible horizontal transfers of *Abermu* sequences between *L. perrieri* and rice. It was reported that a MULE was horizontally transferred between the *Setaria* genus and rice (Diao et al., 2006). We compared *Abermu* transposons with the MULE sequences described by Diao et al. (2006), no significant sequence identity was detected suggesting that they were from different families.

**Table 3:** Comparisons of Ks values of *Abermu* transposons and ADH1 genes.

Species	*Leersia perrieri*	
	*Abermu*	*ADH1*
*Oryza sativa*	1.2346	0.3416
*Oryza nivara*	0.9135	0.3521
*Oryza rufipogon*	6.1059	0.3272
*Oryza glaberrima*	3.7708	0.3430
*Oryza longistaminata*	3.4256	0.3811
*Oryza meridionalis*	2.4485	0.3860
*Oryza punctata*	8.5653	0.3497

### The Origin and Function of ORFR

Except ORF1 encoding mutator transposase, some *Abermu* transposons contain one or two extra ORFs including the ORFR with opposite transcription director (Figure 3). Despite the molecular functions of the two ORFs are not clear, two basic questions need to be addressed. The first question is how and when the ORFR has emerged? As some MULEs (Pack-MULEs) can capture host’s genes (Yu et al., 2000; Jiang et al., 2004; Tan et al., 2021) and the *Abermu* elements in barley and other plants are larger than many MULEs, it was possible that the ORFR sequences were originally from host genomes and have been captured by the old *Abermu* transposons. No sequence similarity was detected between the ORFR sequences identified in distantly related plants. One possibility was that all ORFR sequences in different plants were derived from a single ancestor gene, but they evolved quickly (Table 2). Another possibility was that the ORFRs were acquired independently in different host genomes as we can see variable locations of ORFRs in different *Abermu* transposons, upstream or downstream of the ORF1 (Figure 3). It should be noted that Pack-MULEs are usually small (2–3 Kb) and non-autonomous transposons (Jiang et al., 2004). However, *Hvu*_*Abermu* in barley seems to be an autonomous transposon and has the potential to distribute the ORFR sequences throughout the genome. Second question is regarding the molecular function of ORFRs. In maize, MURB is crucial for the insertion of *MuDR* transposon as no new insertion was found in the lines without the *mudrB* gene (Lisch et al., 1999; Raizada and Walbot, 2000). The proteins encoded by the ORFRs of *Abermu* transposons showed no sequence similarity to MURB. We detected polymorphisms of *Abermu* which contains ORFR in the barley population (Supplementary Table S5). We also conducted genome-wide comparisons of *Osa_Abermu* transposon which lacks the ORFR sequence and identified polymorphic transposons between seven cultivated and wild rice genomes (Data not shown) suggesting recent activity of *Osa_Abermu* and that ORFR may be unimportant for transposition. However, more experiments are needed to better understand the functions of ORFR.

### The Impacts and Potential Applications of *Abermu* Elements

Transposons contribute large fractions of cereal genomes such as 80.8% of the barley genome (Mascher et al., 2017) and 85.0% of the wheat genome (Zhu et al., 2021). However, their impacts on genome evolution and phenotypical mutations in major cereals are not well understood. More recent evidence revealed that transposons played pivotal roles in crop improvement including the seed quality in barley (Lei et al., 2020) and maize domestication (Studer et al., 2011). In the present study, we identified *Abermu* transposons in several agronomically important crops such as barley, wheat, rice, and maize, and found that *Abermu*-related sequences located in over 200 barley genes including CDSs and introns. In some cases, intronic transposons may affect gene functions. For example, a non-LTR retrotransposon located in an intron of the *EgDEF1* gene altered the transcript and generated a truncated peptide causing abnormal fruits in oil palm (Ong-Abdullah et al., 2015). We are not sure if the *Abermu* element inserted in *HORVU.MOREX.r2.1HG0047050.1* (Figure 6) affects the gene expression in barley and the functional divergence of the orthologous genes. The movements of transposons not only increase the host genetic diversity but also provide good resources for marker development. Indeed, our *Abermu*-based markers detected molecular polymorphisms among different barley genotypes (Figure 7).

Active transposons can be used to develop new genetic tools for gene-tagging and other studies (Fernandes et al., 2004). Barley has emerged as a model system for cereal crops, but no active transposon was identified in barley. Thus far, all transposon-based gene-tagging systems in barley were developed with the exogenous Ac/Ds transposons from maize (Singh et al., 2006; Lazarow and Lütticke, 2009). In rice, the endogenous retrotransposon *Tos17* has been widely used for characterizing molecular functions of important genes (Miyao et al., 2003). We identified polymorphic *Abermu* elements within cultivated barley varieties and revealed the recent transpositional activity of *Abermu*. Thus, it provides potential resources for developing gene-tagging systems with this endogenous transposon in barley, wheat, and other crops. However, more experiments are needed to address the value of *Abermu* for gene-tagging system including to investigate if *Abermu* transposon can maintain its transposition activity in different generations and compare the polymorphic insertions between the wild type and the mutants induced by tissue culture and other stresses.

## Data Availability Statement

The datasets presented in this study can be found in online repositories. The names of the repository/repositories and accession number(s) can be found in the article/Supplementary Material.

## Author Contributions

DG and XC designed the experiments. DG and AC performed computational and molecular analysis. HB, GH, and DG identified and provided seeds. DG drafted the manuscript. All authors were involved in manuscript revision and approved the submitted version.

## Conflict of Interest

The authors declare that the research was conducted in the absence of any commercial or financial relationships that could be construed as a potential conflict of interest. Mention of trade names or commercial products in this publication is solely for the purpose of providing specific information and does not imply recommendation or endorsement by the United States Department of Agriculture.

## Publisher’s Note

All claims expressed in this article are solely those of the authors and do not necessarily represent those of their affiliated organizations, or those of the publisher, the editors and the reviewers. Any product that may be evaluated in this article, or claim that may be made by its manufacturer, is not guaranteed or endorsed by the publisher.

## Footnotes

^1^ http://plants.ensembl.org/Hordeum_vulgare/Info/Index

^2^ http://rice.uga.edu

^3^ http://www.repeatmasker.org

^4^ https://bedtools.readthedocs.io

^5^ https://www.sanger.ac.uk/tool/artemis-comparison-tool-act
